# Antibacterial Peptide BSN-37 Kills *Extra*- and *Intra*-Cellular *Salmonella enterica* Serovar Typhimurium by a Nonlytic Mode of Action

**DOI:** 10.3389/fmicb.2020.00174

**Published:** 2020-02-07

**Authors:** Lei Yang, Yawei Sun, Yanzhao Xu, Bolin Hang, Lei Wang, Ke Zhen, Bing Hu, Yanan Chen, Xiaojing Xia, Jianhe Hu

**Affiliations:** College of Animal Science and Veterinary Medicine, Henan Institute of Science and Technology, Xinxiang, China

**Keywords:** antibacterial peptide BSN-37, *Salmonella enterica* serovar Typhimurium, antibacterial activity, action mode, eukaryotic cells, cytotoxicity

## Abstract

The increasing rates of resistance to traditional anti-*Salmonella* agents have made the treatment of invasive salmonellosis more problematic, which necessitates the search for new antimicrobial compounds. In this study, the action mode of BSN-37, a novel antibacterial peptide (AMP) from bovine spleen neutrophils, was investigated against *Salmonella enterica* serovar Typhimurium (*S.* Typhimurium). Minimum inhibitory concentrations (MICs) and time-kill kinetics of BSN-37 were determined. The cell membrane changes of *S.* Typhimurium CVCC541 (ST) treated with BSN-37 were investigated by testing the fluorescence intensity of membrane probes and the release of cytoplasmic β-galactosidase activity. Likewise, cell morphological and ultrastructural changes were also observed using scanning and transmission electron microscopes. Furthermore, the cytotoxicity of BSN-37 was detected by a CCK-8 kit and real-time cell assay. The proliferation inhibition of BSN-37 against intracellular *S.* Typhimurium was performed in Madin-Darby canine kidney (MDCK) cells. The results demonstrated that BSN-37 exhibited strong antibacterial activity against ST (MICs, 16.67 μg/ml), which was not remarkably affected by the serum salts at a physiological concentration. However, the presence of CaCl_2_ led to an increase in MIC of BSN-37 by about 4-fold compared to that of ST. BSN-37 at the concentration of 100 μg/ml could completely kill ST after co-incubation for 6 h. Likewise, BSN-37 at different concentrations (50, 100, and 200 μg/ml) could increase the outer membrane permeability of ST but not impair its inner membrane integrity. Moreover, no broken and ruptured cells were found in the figures of scanning and transmission electron microscopes. These results demonstrate that BSN-37 exerts its antibacterial activity against *S.* Typhimurium by a non-lytic mode of action. Importantly, BSN-37 had no toxicity to the tested eukaryotic cells, even at a concentration of 800 μg/ml. BSN-37 could significantly inhibit the proliferation of intracellular *S.* Typhimurium.

## Introduction

*Salmonella enterica* serovar Typhimurium (*S.* Typhimurium) is an important etiological agent responsible for invasive salmonellosis, which can be treated with antibacterial agents. However, the appearance and spread of multidrug-resistant *S.* Typhimurium has created a clinical dilemma for the treatment of invasive salmonellosis (Crump et al., 2015; Balasubramanian et al., 2019). Therefore, searching for new antibacterial compounds to treat invasive salmonellosis has become inevitable and urgent. Antimicrobial peptides (AMPs) are plausible candidates for the design of new antibacterial agents.

Antimicrobial peptides in living organisms are important components of innate immunity to microbial infections (Spänig and Heider, 2019). More than 3,000 AMP sequences have been registered in the antibacterial peptide database^1^. Among them, cathelicidins are one of the best characterized AMP families. Each cathelicidin includes an N-terminal signal domain, a cathelin-like domain, and an antimicrobial C-terminal domain. To defend against microbial attack *in vivo*, C-terminal domains in the AMPs are released and become mature peptides that exhibit biological activities. The proline-rich peptides (PRPs) are important members of the cathelicidin family, which contain high numbers of proline and arginine in mature sequences. In recent years, the PRPs have been attracting the close attention of researchers due to its strong antibacterial activity against Gram-negative bacteria, remarkably low toxicity toward mammalian cells, and the lack of extensive membrane-damaging effects (Ramanathan et al., 2002; Mishra et al., 2018). Bac5 is the first member of the PRPs among the cathelicidins family which demonstrated effective antibacterial activity against *Escherichia coli* ATCC 25922, *S.* Typhimurium LT2, *S.* Typhimurium ATCC14028, and *Klebsiella pneumoniae* ATCC1388 (MICs, 12–25 μg/ml). Moreover, Bac5 at the concentration of 50 μg/ml can completely kill *S.* Typhimurium ATCC14028 after co-incubation for 60 min (Gennaro et al., 1989). Active Bac5 is comprised of 42 amino acid residues with a repeated pattern of Arg-Pro-Pro triplets. Since Bac5 lacks any cysteine residue, it cannot form a disulphide bridge and exists in a linear conformation (Price et al., 2019). Research has found that the presence of Arg residues at or near the N-terminus and a chain length exceeding 15 residues, are required for the truncated fragments to exhibit antibacterial activity to *E. coli* IFO 12743, *Staphylococcus aureus* IFO12732, and *Bacillus subtilis* IFO 3134. When the first four residues in the N-terminus of Bac5(1-23) were deleted, Bac5(4-23) lost antibacterial activity (Tokunaga et al., 2001). Bac5(1-25) and Bac5(1-31) entered *E. coli* BW25113 mainly through the inner membrane transport proteins, SbmA. In the cytoplasm, the two peptides prohibited the protein from synthesizing by directly binding to the peptide exit tunnel in the 70S ribosomes. However, the inhibition ability of Bac5(1-25) and Bac5(1-31) seems to be species-specific due to the low affinity exhibited for *Thermus thermophilus* 70S ribosomes (Mardirossian et al., 2018). Furthermore, Bac5(1-25) was the best truncated fragment according to a comprehensive evaluation of antibacterial activity, cytotoxicity, and inhibition efficacy of protein synthesis (Mardirossian et al., 2019).

The Bac5 pre-pro peptide is actively synthesized in immature myeloid cells and comprises a 29-amino acid signal peptide followed by a 101-amino acid pro-region. After removal of the signal peptide, ProBac5 is stored in large cytoplasmic granules of bovine neutrophils (Zanetti et al., 1990). When peripheral neutrophils are activated, ProBac5 is secreted and forms the active AMP after the digestion of elastase from the azurophils (Scocchl et al., 1992). At present, the large granules have only been reported in the neutrophils of cow, goat, sheep, and deer, in which the Bac5 orthologs (QaBac5α, ChBac5, and P9) were also found (Shamova et al., 1999; Treffers et al., 2005). In the chromosome of sheep, four genes encoding PRPs (QaBac5, QaBac6, QaBac7.5, and QaBac11) have been identified. A naturally active C-terminal fragment of QaBac7.5 [QaBac7.5 mini (32–60)] and different naturally active N-terminal fragments of QaBac11 were purified from ovine neutrophil extract. The researchers speculated that these truncated active PRPs fragments might come from the cleavage of parent PRPs digested by peptidases (Anderson and Yu, 2003). Additionally, ChBac7Nα(1-21) and ChBac7Nβ(1-22), two highly active N-terminal fragments of PRP ChBac7.5, were also isolated from goat neutrophils. It was assumed that the fragmentation of mature cathelicidin-related AMPs may play a key role in different types of defense responses (Shamova et al., 2016). Except for the naturally active fragments of Bac7.5, three variants of QaBac5 (QaBac5α, QaBac5β, and QaBac5γ) and an N-terminal variant of ChBac5 (ChBac3.4) were also isolated from sheep and goat, respectively, which showed antibacterial activity to the tested bacteria (Anderson and Yu, 2003; Shamova et al., 2009). However, no naturally active truncated fragment of Bac5 was reported until we found BSN-37, a truncated N-terminal fragment [Bac5(2-38)] of Bac5, from bovine spleen neutrophils in 2018 (Yang et al., 2018). Additionally, the action model of Bac5 and its truncated fragments against *S.* Typhimurium is not very clear.

Our previous experiments showed that BSN-37 exhibited good antibacterial activity to susceptible and multidrug-resistant *S.* Typhimurium and *E. coli* (MICs, 3.13–50 μg/ml). Moreover, BSN-37 at the concentration of 400 μg/ml exhibited low hemolytic rates (≤1.34%) and had no toxicity to the tested eukaryotic cells. In this experiment, we further confirmed that BSN-37 killed *S.* Typhimurium by a non-lytic mode of action and could significantly inhibit the proliferation of intracellular *S.* Typhimurium.

## Materials and Methods

### Antimicrobial Peptides and Antibiotics

Two peptides, BSN-37 (N-FRPPIRRPPIRPPFYPPFRPPIRPPIF PPIRPPFRPP-C) and pexiganan (N-KKLIKVFAKGFKKAKKLF KGIG-C), were prepared via solid-phase synthesis using 9-fluorenylmethoxycarbonyl (F-moc) chemistry at GL Biochem Ltd., (Shanghai) and analyzed by HPLC and MALDI-TOF MS to confirm that the purity was >95%. Pexiganan is a synthetic analog of the peptide magainin II from the skin of the African clawed frog, *Xenopus laevis*, which exhibits a lytic mode of action against bacteria (Gottler and Ramamoorthy, 2009), and is used as a positive control in this study. Both peptides were dissolved in ultrapure water and used for the following experiments.

Amoxicillin and chloramphenicol were purchased from the National Institute for the Control of Pharmaceutical and Biological Products (Beijing, China).

### Bacterial Strains and Cells

The strains of *E. coli* ATCC25922 and *Staphylococcus aureus* ATCC25923 were purchased from the American Type Culture Collection (ATCC, Manassas, VA, United States). The strain of *S.* Typhimurium CVCC541 was purchased from the China Institute of Veterinary Drug Control (CVCC, Beijing, China) and designated ST in this article. The strains of *S.* Typhimurium CMCC50097 and *Staphylococcus albus* CMCC261015 were purchased from the National Center for Medical Culture Collections (CMCC, Beijing, China). The strain of *Salmonella pullorum* BNCC124693 was purchased from the BeNa Culture Collection (BNCC, Suzhou, China). A clinical isolate of *S.* Typhimurium SB217 from a diseased chicken was identified by the VITEK-32 system (bioMérieux) and PCR amplification sequencing.

Porcine small intestinal epithelial cells (IPEC-J2), Vero cells, and Madin-Darby canine kidney (MDCK) epithelial cells were purchased from the American Type Culture Collection (ATCC, Manassas, VA, United States).

### Antimicrobial Measurements and Salt Sensitivity Assay

Minimum inhibitory concentrations (MICs) of BSN-37, pexiganan, amoxicillin, and chloramphenicol were determined using the two-fold broth microdilution method according to the CLSI guidance (Clinical and Laboratory Standards Institute [CLSI], 2017). MIC values of the tested antibacterial agents were determined on three independent occasions.

Next, the effect of different salts on the antibacterial activity of BSN-37 was tested as described by Maisetta et al. (2018). MICs of BSN-37 against *E. coli* ATCC25922 and ST were measured in the presence of physiological concentrations of salts (150 mM NaCl, 4.5 mM KCl, 6 μM NH_4_Cl, 1 mM MgCl_2_, 2.5 mM CaCl_2_, and 4 μM FeCl_3_).

### Time-Kill Kinetics Curve

Time-kill kinetics of BSN-37 against ST was determined according to the following method. A solution of equal parts ST at a concentration of 5 × 10^5^ CFU/ml in fresh Mueller-Hinton (MH) broth and BSN-37 at different concentrations (0, 100, 200, and 400 μg/ml) were prepared and grown at 37°C without shaking. Each sample (100 μl) was withdrawn at 0, 1, 2, 4, 6, 8, and 24 h and serially diluted in Luria-Bertani (LB) broth by a 10-fold decrease. The diluted bacteria were plated onto LB agar plates and cultured at 37°C for 18 h for the colony-forming unit (CFU) counts.

### Outer Cell Membrane Permeability

The fluorescence of N-phenyl-1-naphthylamine (NPN) is weak in an aqueous environment but strong in non-polar or hydrophobic environments. In normal bacteria NPN is excluded from the outer cell membrane and does not fluoresce. If the outer membrane of bacteria is damaged, NPN partitions into the interior of the outer cell membrane, and an increase in fluorescence can be observed. Therefore, the outer membrane permeability of BSN-37 and pexiganan against ST was evaluated using an NPN uptake assay (Helander and Mattila-Sandholm, 2000). An overnight culture of 50 μl was added into 5 ml fresh LB and grown at 37°C with shaking at 250 rpm to an optical density (OD) at 600 nm of 0.4 to 0.6. The strains were harvested by centrifugation at 5000 × *g* for 6 min and then washed twice in 5 mM HEPES buffer containing 5 mM glucose (working solution, pH 7.4). The bacterial sediment was resuspended in the working solution at an OD_600_ = 1. Further, 100 μl bacteria suspension, 50 μl working solution containing 40 μM NPN, and 50 μl peptide at different concentrations were added to a sterile 96-well plate and mixed. Fluorescence was monitored immediately in a multifunctional microplate reader (Infinite^®^ 2000, TECAN) at an excitation wavelength of 350 nm and an emission wavelength of 420 nm for 1 h at 1 min intervals.

### Inner Membrane Integrity

The fluorescent probe, 3,3′-dipropylthiadicarbocyanine iodide [DiS-C3-(5)], is concentrated in the cytoplasmic membrane under the influence of the internally negative membrane potential, leading to self-quenching of the fluorescence. If peptides form channels or otherwise disrupt the membrane, the membrane potential will be dissipated, and the DiS-C3-(5) will be released into the medium, causing the fluorescence to increase. Therefore, the inner membrane integrity of ST treated with BSN-37 and pexiganan was tested using DiS-C3-(5) as a probe (Sautrey et al., 2016). ST was grown to the mid-logarithmic phase (OD_600_ = 0.5–0.6) in LB broth and harvested by centrifugation at 5000 × *g* for 6 min. The cells were washed twice in a working solution and then resuspended in the same solution at an A_600_ = 0.05. After that, DiS-C_3_-(5) at the final concentration of 0.8 mM was added into the suspension. At the DiS-C_3_-(5) uptake maximum, 100 mM KCl was added to equilibrate the cytoplasmic and external K^+^ concentration. After incubation in the dark for 1 h at room temperature, each 180 μl cell suspension was transferred to the wells supplied with 20 μl BSN-37 at different concentrations on a 96-well black polystyrene microtiter plate (Corning, NY, United States). Congruently, pexiganan at the final concentration of 100 μg/ml was used as a control. Fluorescence intensity was monitored in a multifunctional microplate reader (Infinite^®^ 2000, TECAN) at an excitation wavelength of 620 nm and an emission wavelength of 670 nm for 30 min at 1 min intervals. Each experiment was repeated three times.

### β-Galactosidase Assay

**FIGURE 1:**
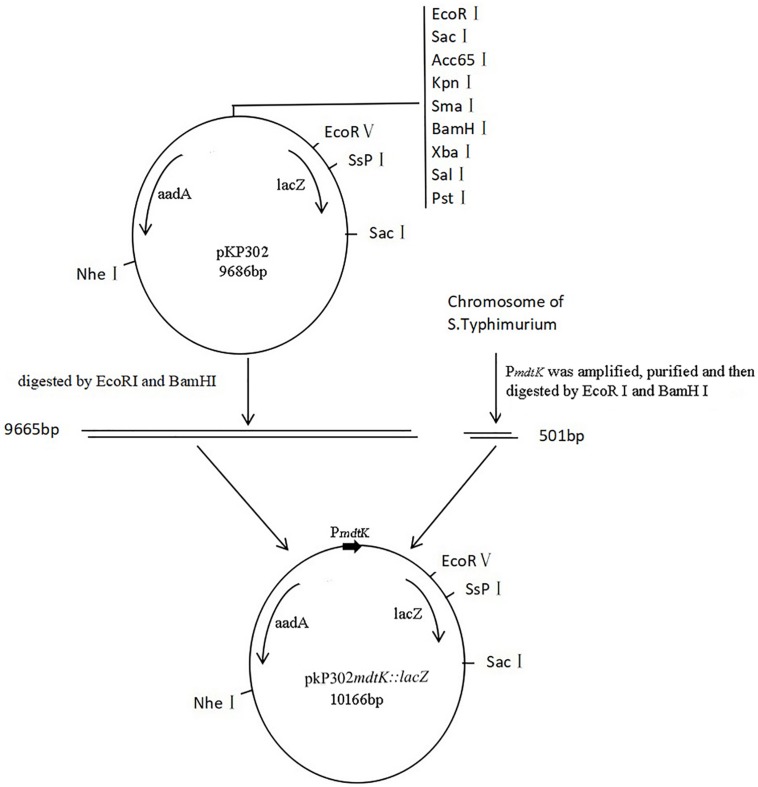
Construction of recombined plasmid pKP302*mdtK:lacZ* P*mdtK* was amplified from *mdtK* locating in the chromosome of *S.* Typhimurium and purified. The products of P*mdtK* digested by EcoRI and BamHI were ligated to the fragments of plasmid pKP302 digested with the same enzymes. The recombined plasmids were transformed into DH5α and then, identified by PCR. *lacZ*, a gene encoding β-galactosidase; *aadA*, a gene encoding resistance to streptomycin and spectinomycin; P*mdtK*, the promoter region of *mdtK*.

To determine if BSN-37 disrupts the inner cell membrane, the release of cytoplasmic β-galactosidase activity was measured in ST containing a fusion plasmid pKP302*mdtk:lacZ* using onitrophenyl-β-D-galactopyranoside (ONPG, Sigma, Germany) as a substrate according to a previously described method (Paytubi et al., 2017). Due to the deficiency of gene encoding β-galactosidase in ST, a fusion plasmid pKP302*mdtk:lacZ* was first constructed according to the method shown in Figure 1. The primer set used to amplify the promoter region of the multidrug efflux pump, MdtK, in ST was KLAF 5′-CCGAATTCGGTGCGAAAG-3′ and KLAR 5′-TTGGATCCGCCAGCAGTAA-3′ (restriction sites are underlined). The primer set used to identify the fusion plasmid was PKPF 5′-TGCTCCATAACATCAAA CATCG-3′ and PKPR 5′-GCATAACCACCACGCTCATC-3′, which was located on the two sides of the inserted fragment in pKP302. Finally, the fusion plasmid pKP302*mdtk:lacZ* was transformed into ST by electroporation. Secondly, a β-galactosidase assay was performed. Briefly, overnight cultures of STpKP302 *mdtk:lacZ* were diluted (1:100) in LB medium containing 100 μg/ml spectinomycin and grown at 37°C with shaking at 250 rpm to an OD_600_ = 0.3–0.5. The OD_600_ value was recorded. A culture of 200 μl was pelleted and resuspended in 400 μl Z-buffer (0.06 M Na_2_HPO_4_, 0.04 M NaH_2_PO_4_, 0.01 M KCl, 0.001 M MgSO_4_, and 0.05 M β-mercaptoethanol, pH 7.0). After that, 20 μl of chloroform and 10 μl of SDS 0.1% (w/v) were added, and the mixture was intensively vortexed for 15 s. The sample was used as a positive control. Alternatively, each 200 μl culture sediment was individually resuspended in 400 μl PBS buffers (10 mM) supplied with different concentrations of BSN-37 (0, 50, 100, and 200 μg/ml). All samples were incubated at 30°C for 1, 2, 4, or 6 h. At each incubation ends, the reaction was initiated by the addition of 80 μl ONPG (4 mg/ml). Once the reaction began to turn yellow, it was quenched by the addition of 200 μl sodium carbonate (1 M). After 5 min, OD_420_ nm and OD_550_ nm of the reaction mixtures were determined, and β-galactosidase activity (Miller Units) was calculated using the following formula: Miller Units = 1000 × (OD_420_−1.75 × OD_550_)/[volume (ml) × time (min) × OD_600_]. Enzymatic determinations were performed in duplicates of at least three independent experiments.

### Scanning Electron Microscopy

To reduce the effect of the centrifugation on the morphology of the bacteria, an improved SEM operation procedure was followed as described below. Sterile slides were placed into 24-well plates with 1 ml MH broth in each well. An overnight culture of 10 μl ST was added into each well and cultured at 37°C for 4 h. After incubation, the culture medium was replaced by 1 ml BSN-37 at different concentrations (50, 100, and 200 μg/ml) and reacted for 1 h. Alternatively, the slide treated with 50 μg/ml pexiganan and 10 mM PBS buffer was used as a positive and negative control, respectively. After the slides in the wells were gently washed twice with warm PBS buffer, 400 μl glutaraldehyde (2.5%, v/v) was added into each well to fix bacteria at 4°C overnight. The fixed bacteria were dehydrated for 15 min in a series of graded ethanol solutions (30, 50, 70, 80, 90, and 100%). The samples were transferred to a mixture (1:1) of ethanol and tertiary butanol for 15 min and then to pure tertiary butanol for 20 min. After lyophilization and gold coating, the morphology of the bacteria was observed by SEM under a Quanta 200 FEG scanning electron microscope (FEI, Ltd.).

### Transmission Electron Microscope

Transmission Electron Microscope imaging was performed as described previously (Shao et al., 2018). The working concentration of BSN-37 and pexiganan with cell suspensions was 200 and 50 μg/ml, respectively. Finally, the specimens were sectioned with an ultramicrotome, stained with uranyl acetate and lead citrate, and observed using a Hitachi HT-7770 TEM (Hitachi, Japan).

### CCK-8 Assay

The toxicity of BSN-37 and pexiganan against IPEC-J2 cells was determined using a CCK-8 assay kit (CCK-8, Dojindo, Kumamoto, Japan). IPEC-J2 cells were cultured in Advanced DMEM/F12 medium (Gibco, Thermo Fisher Scientific, United States) supplied with 0.2 mM L-glutamine (Gibco, Thermo Fisher Scientific, United States) and 5% fetal bovine serum (Gibco, Thermo Fisher Scientific, United States) at 37°C in an atmosphere containing 5% carbon dioxide with a relative humidity of 86%. Cells at approximately 2 × 10^5^ cells/ml were seeded into 96-well culture plates (100 μl/well). Peptides (100 μl) at different concentrations (50, 100, 200, and 400 μg/ml) were added into the wells. The wells containing cells without peptides served as controls. After incubating the plates for 24 h, 10 μl CCK-8 solution was added, the incubation continued for 2 h at 37°C, and the absorbance at 450 nm was read with the aid of a multifunctional microplate reader (Infinite^®^ 2000, TECAN). The viability of the control cells was assumed to be 100%. The viability of cells exposed to peptides was expressed as percentages of the control. This assay was performed in triplicate.

### Real-Time Cell Assay

The xCELLigence system (ACEA Biosciences; San Diego, CA, United States) was used to detect IPEC-J2 cells and Vero cells proliferation according to a previously described method (Pauly et al., 2012). Cells seeded at 7500 cells/well and 10000 cells/well in 16-well E-plates were cultured to ∼60% confluency based on cell index (CI) values. Various concentrations of BSN-37 (50, 100, 200, 400, and 800 μg/ml) were added to the treated cells and were incubated for 40 h. Congruently, the pexiganan at a concentration of 100 μg/ml and the cells without AMP were used as a positive and negative control, respectively. The CI was automatically determined every 15 min by the xCELLigence system (ACEA Biosciences; San Diego, CA, United States). In the assay process, each sample (100 μl) from the BSN-37(100 μg/ml)-treated group and the positive control was withdrawn at random four times sites and used for the CFU counts. The cells’ survival rates were decided and analyzed.

### *In vitro* Proliferation Inhibition of Intracellular *Salmonella* Assay

To investigate the effect of BSN-37 on the proliferation of intracellular bacteria, a gentamicin protection assay was performed as previously described (Gerlach et al., 2008). In this experiment, ST and SB217 were used as invasive strains. MDCK cells (1 × 10^5^ cells/well) were plated on 24-well plates for 24 h. Cells were infected by ST or SB217 at a multiplicity of infection (MOI) of 10∼100:1 to host cells. Cells were incubated with bacteria for 1 h at 37°C, followed by 2 h of incubation in media containing 100 μg/ml gentamicin at 37°C to kill extracellular bacteria. The infected cells were divided into three groups. The first group was washed twice with warm PBS buffer and then lysed by adding 200 μl of 1% Triton-100 for 10 min. The lysate was used for the CFU counts. Invasion ability of the tested strains is expressed as a percentage of initial bacterial inoculum. The second and third groups were treated with 0 and 400 μg/ml of BSN-37, respectively, in the presence of 10 μg/ml gentamicin for 14 h at 37°C. After that, each group was also treated using the same method to that in the first group. Fold intracellular replication was calculated by dividing the intracellular bacterial load at 16 h by the intracellular bacterial load at 2 h. Each experiment was performed three times.

### Statistical Analysis

Statistical analyses were performed using GraphPad Prism 6.0 (GraphPad Software). Two-way ANOVA or unpaired Student’s *t*-test were utilized where appropriate. The threshold of statistical significance was set at ^∗^*P* < 0.05, ^∗∗^*P* < 0.01, ^∗∗∗^*P* < 0.001, and ^****^P < 0.0001. Exact *P*-values are indicated in the figure legends, when applicable.

## Results

### Antibacterial Activity of BSN-37 and Serum Salts Effect

The MICs of amoxicillin, chloramphenicol, BSN-37, and pexiganan against four typical Gram-negative and two Gram-positive bacteria are shown in Table 1. BSN-37 showed antibacterial activity to the tested strains (MICs ≤ 50 μg/ml) except for *S. aureus* ATCC25923. Among them, BSN-37 exhibited strong inhibitory action against *S.* Typhimurium. The MICs of BSN-37 against ST and *S.* Typhimurium CMCC50097 are 16.67 and 8.33 μg/ml, respectively.

**TABLE 1:** Minimum inhibitory concentrations of the strains to antibiotics and antimicrobial peptides.

Strains	MIC(μg/ml)			
	
	AMO	CHL	BSN-37	Pexiganan
*E. coli* ATCC25922	8	3.13	33.33	10.41
ST	0.83	1.67	16.67	6.25
*S. enterica* serovar Typhimurium CMCC50097	2.67	2.67	8.33	6.25
*S. pullorum* BNCC124693	1.67	4	25	50
*S. aureus* ATCC25923	0.67	13.33	>200	20.83
*S. albus* CMCC26101	0.5	16	50	10.45

*AMO, amoxicillin; CHL, chloramphenicol; ST, *S. enterica* serovar Typhimurium CVCC541.*

In general, the serum salts probably affected the antibacterial activity of AMPs *in vivo*. The MICs of BSN-37 against ST and *E. coli* ATCC25922 in the presence and absence of different serum salts at physiological concentrations are shown in Table 2. The MICs of BSN-37 against ST did not exhibit significant changes (<1.5-fold increase) in the presence of the tested salts, except that CaCl_2_ led to MICs increase of about 4-fold. In contrast, the antibacterial activity of BSN-37 shows strong inhibition against *E. coli* ATCC25922 with NaCl, MgCl_2_, or CaCl_2_, which decreased its activity at least 6-fold. The remaining salts, KCl, NH_4_Cl, and FeCl_3_, did not significantly affect the antibacterial activity of BSN-37 against *E. coli* ATCC25922.

**TABLE 2:** Minimum inhibitory concentrations of BSN-37 in the presence and absence of physiological concentrations of different serum salts.

Culture medium	Strains (unit: μg/ml)	
	
	*E. coli* ATCC25922	ST
MH broth + NaCl (150 mM)	200	20.83
MH broth + KCl (4.5 mM)	50	16.67
MH broth + NH_4_Cl (6 μM)	50	16.67
MH broth + MgCl_2_ (1 mM)	200	25
MH broth + CaCl_2_ (2.5 mM)	>200	66.67
MH broth + FeCl_3_ (4 μM)	50	12.5
MH broth	33.33	16.67

*ST, *S. enterica* serovar Typhimurium CVCC541.*

### Bactericidal Kinetics of BSN-37

**FIGURE 2:**
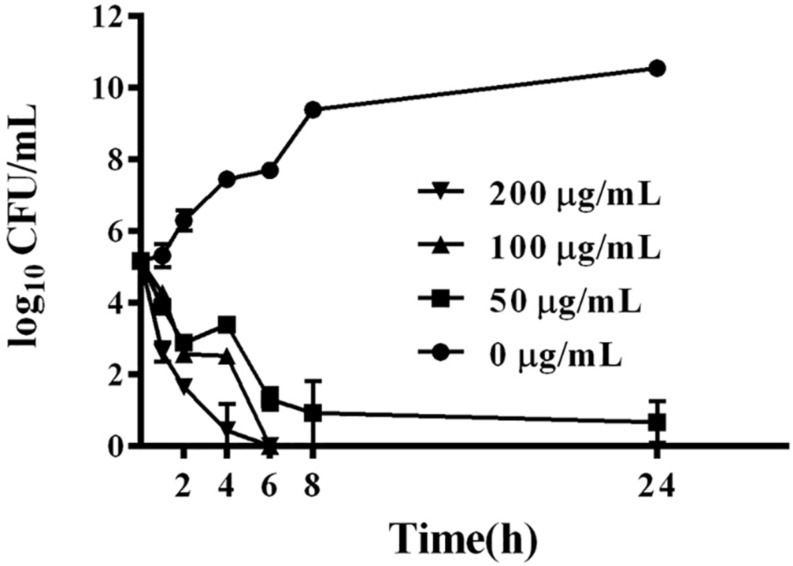
Time-dependent killing curves of ST incubated with BSN-37 at different concentrations of BSN-37 (0, 50, 100, and 200 μg/ml) in MH broth medium for 24 h. Aliquots were collected at 0, 1, 2, 4, 6, 8, and 24 h to count the bacteria. Error bars represent means ± SD. Assays were performed in triplicate.

BSN-37 exhibited strong bacteriostatic action against *S.* Typhimurium; therefore, the bactericidal kinetics of BSN-37 against ST was further studied by determining the time-kill curve. As shown in Figure 2, BSN-37 at the concentration of 100 or 200 μg/ml completely killed the strain after co-incubation for 6 h. However, BSN-37 at the concentration of 50 μg/ml did not completely kill the strain after co-incubation for 24 h, although the concentration of the bacteria reduced from 1.45 × 10^5^ to 8.43 CFU/ml in the first co-incubation for 8 h. Likewise, BSN-37 exhibited concentration- dependent bactericidal action against ST in the co-incubation from 2 h to 6 h.

### Effect of BSN-37 on the Cell Membrane of ST

**FIGURE 3:**
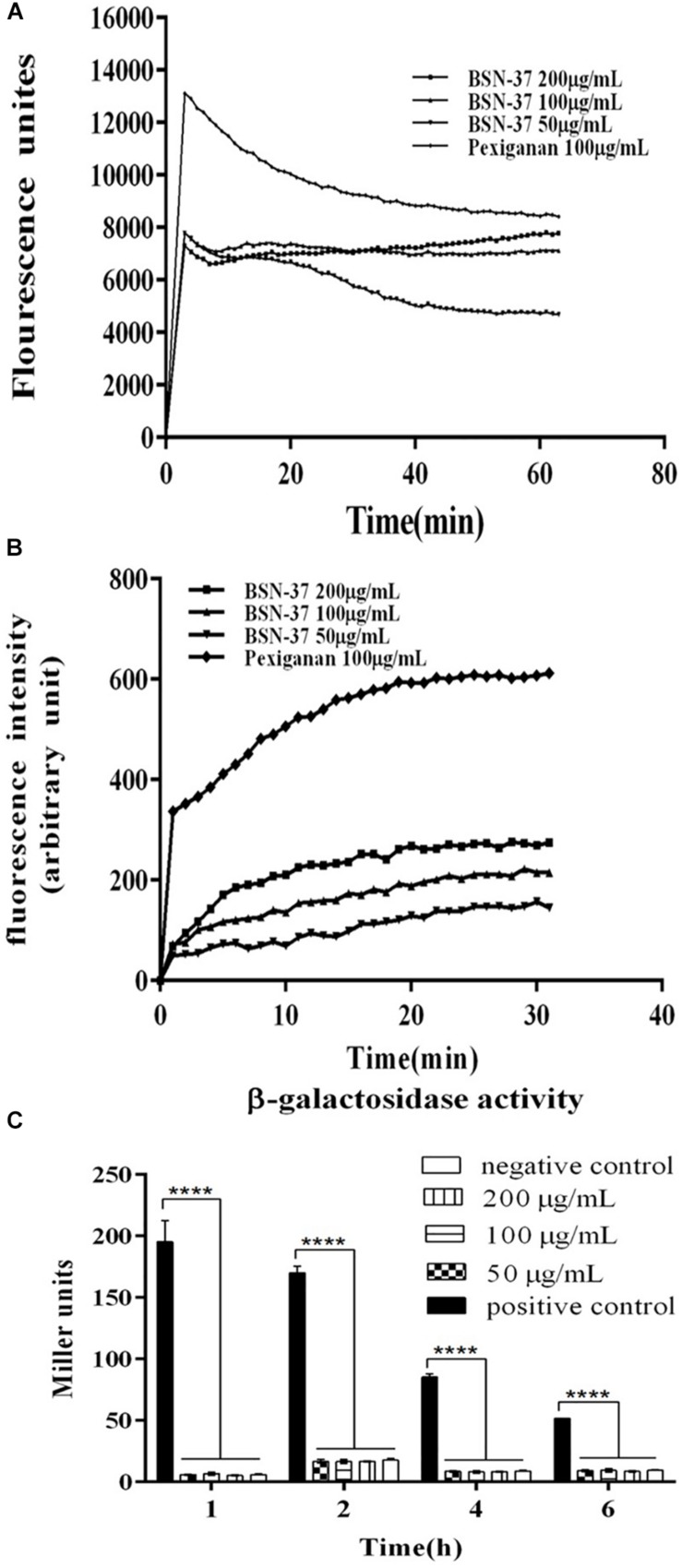
The effect of BSN-37 at different concentrations (50, 100, and 200 μg/ml) and pexiganan at the concentration of 100 μg/ml on the outer membrane permeabilization and inner membrane integrity of ST. **(A)** Time–response curve of the outer membrane permeation of ST cells to NPN. **(B)** Time–response curve of the inner membrane Integrity of ST cells to DiS-C_3_-(5) **(C)** The β-galactosidase activity in the culture medium of STpKP302*mdtk:lacZ* cells treated with BSN-37 and permeabilization mixture. STpKP302*mdtk:lacZ* cells treated with permeabilization mixture containing Z-buffer, chloroform and sodium dodecyl sulfate and PBS buffer was used as positive and negative control, respectively.

The interactions of BSN-37 and pexiganan with the outer membrane of ST were determined by testing the fluorescence of membrane probe NPN. As shown in Figure 3A, the fluorescence units of the sample treated with 100 μg/ml pexiganan significantly increased in the first incubation for about 3 min to 13 000 units. At the same time, the fluorescence units of the samples treated with BSN-37 at different concentrations also showed rapid increases in the first incubation for about 2 min. The max fluorescence unit is about 0.6-fold of that produced by pexiganan. The rapid uptake of NPN demonstrates that BSN-37 and pexiganan can increase the outer membrane permeability of ST, and the efficiency of BSN-37 is weaker than that of pexiganan.

As shown in Figure 3B, pexiganan induced a large release of DiS-C_3__–_(5) (about 320AU) in the first incubation for about 2 min. However, BSN-37 at different concentrations only induced a little release of DiS-C_3__–_(5) (about 50 AU) during a similar incubation time, and the release is concentration-independent, which suggests that BSN-37 has little effect on the inner membrane integrity of ST.

When STpKP302*mdtk:lacZ* was treated with a permeabilization mixture containing Z-buffer, chloroform, and sodium dodecyl sulfate at different times, the β-galactosidase was immediately released (shown in Figure 3C, positive control). However, when STpKP302*mdtk:lacZ* was treated by BSN-37 at different concentrations and times, the β-galactosidase activities in the culture mediums did not exhibit a significant difference compared to that in the negative control (PBS buffer), which demonstrates that BSN-37 does impair the inner membrane integrity of ST.

### SEM and TEM

**FIGURE 4:**
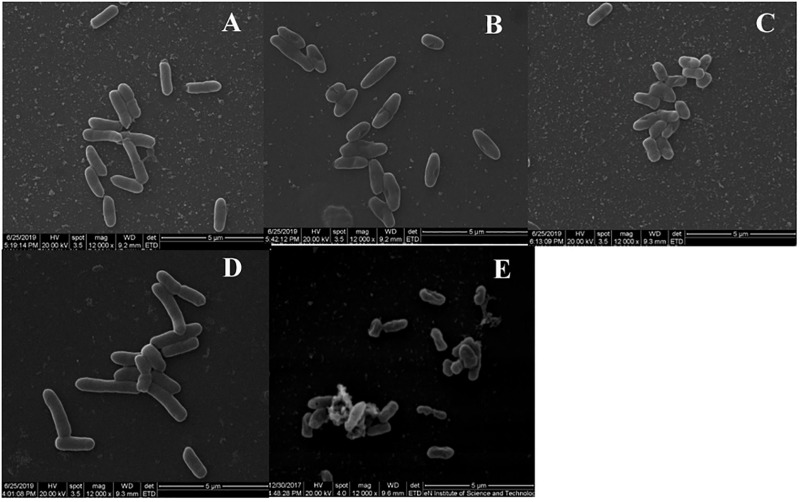
Scanning electron microscropy images of ST treated with BSN-37 and PG for 1 h. **(A)** ST treated with 50 μg/ml BSN-37; **(B)** ST treated with 100 μg/ml BSN-37; **(C)** ST treated with 200 μg/ml BSN-37; **(D)** ST treated with 10 mM PBS buffer; **(E)** ST treated with 50 μg/ml pexiganan.

**FIGURE 5:**
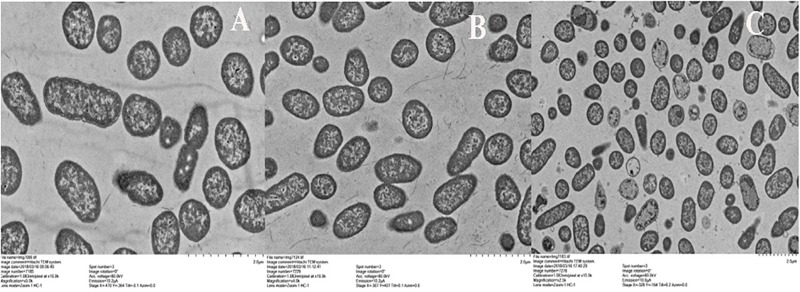
Transmission electron microscropy images of ST treated with BSN-37 and PG for 1 h. **(A)** ST treated with 10 mM PBS buffer; **(B)** ST treated with 200 μg/ml BSN-37; **(C)** ST treated with 50 μg/ml pexiganan.

To observe the effect of BSN-37 on the morphological and ultrastructural changes of ST, SEM, and TEM were used. As shown in Figure 4, the morphology of ST treated with 50 μg/ml BSN-37 was similar to the smooth and regular surface of the control treated with 10 mM PBS. When cells were treated with 100 μg/ml BSN-37, one or two umbilications of the cell membrane in the middle of each bacterium were observed but no broken and ruptured cells were found. Even ST exposed to 200 μg/ml BSN-37 remained unlysed and showed obvious shrinkage in size regardless of the umbilications of the cell membrane. In contrast, atrophy, corrugation and pore-forming were observed on the surface of the cell membrane of ST treated with 50 μg/ml pexiganan. Simultaneously, some intracellular materials leaked from the cell membrane, which formed aggregations and adhesions. Conversely, the TEM figures showed no significant change in morphology and cytoplasm structure of ST treated with 200 μg/ml BSN-37 when compared to that of the negative control (shown in Figure 5). However, morphological changes, substantial plasmolysis, the release of intracellular materials, and bacterial ghosts existed after ST was treated with 50 μg/ml pexiganan. In conclusion, these results concur with the changes of the cell membrane, which demonstrates that BSN-37 does not lyse ST at the tested concentrations.

### Cytotoxicity of BSN-37

**FIGURE 6:**
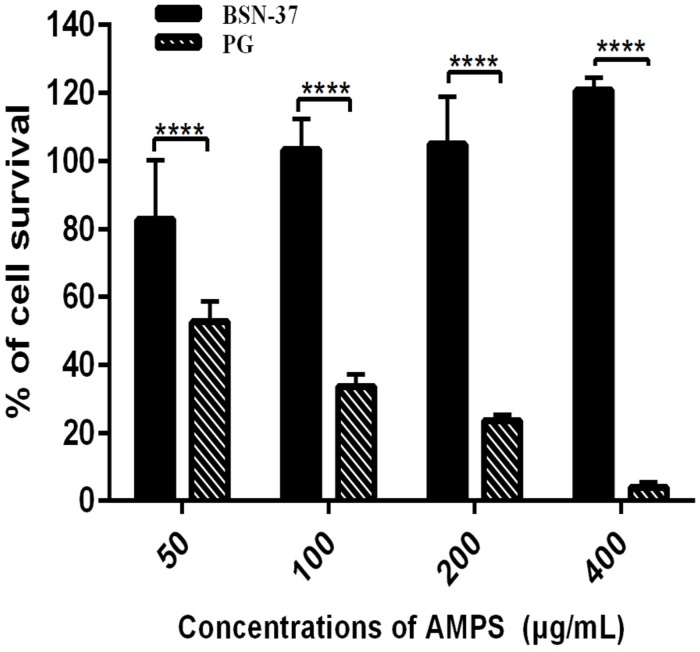
The survival rates of IPEC-J2 cells treated with BSN-37 and PG at a different concentrations by CCK-8 assay. Error bar represents mean ± SEM, *n* = 3. **** represent extremely significance (*P* < 0.0001 by two-way ANOVA).

The toxicity of BSN-37 to IPEC-J2 cells and Vero cells was investigated using the CCK-8 method and real-time cell analysis via the xCELLigence system. In the CCK-8 assay, the survival rate of IPEC-J2 cells treated with BSN-37 at the concentration of 50 μg/ml was 82.74%. The survival rates were all greater than 100% when the concentrations of BSN-37 were 100, 200, and 400 μg/ml. As a positive control group, pexiganan showed concentration-dependent cytotoxicity. The survival rate of IPEC-J2 cells was 52.64 and 3.98% in pexiganan at the concentration of 50 and 400 μg/ml, respectively, (shown in Figure 6).

**FIGURE 7:**
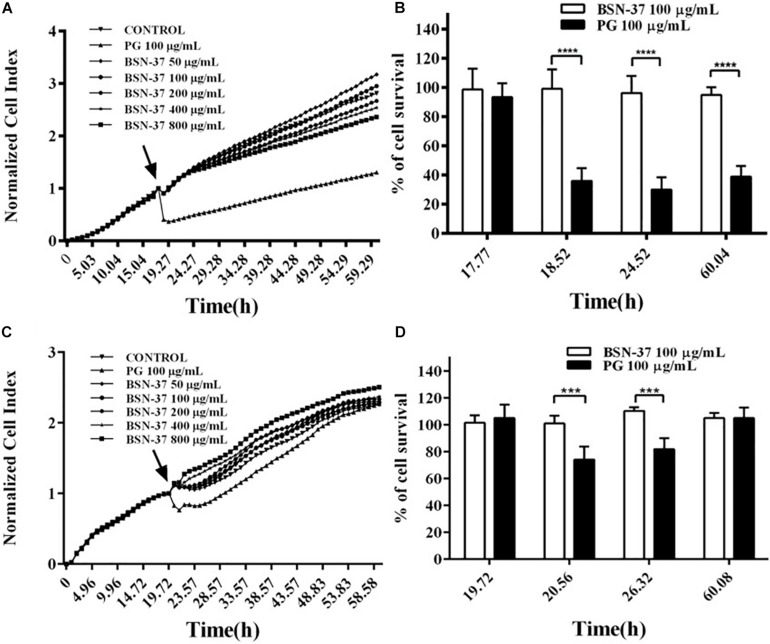
Real-time measurement of the cytotoxicity of BSN-37 and pexiganan against IPEC-J2 cells and Vero cells and the survival rates of IPEC-J2 cells and Vero cells incubated with 100 μg/ml BSN-37 and 100 μg/ml pexiganan for different times. **(A,C)** Real-time monitoring of the cytotoxicity of IPEC-J2 cells and Vero cells, respectively, treated with BSN-37 at a different concentrations (50, 100, 200, 400, and 800 μg/ml) and pexiganan at the concentration of 100 μg/ml for 60 h using the xCELLigence system. The black arrows indicate the time points of adding the peptides. The cells without antimicrobial peptide were used as control. **(B,D)** parallel comparisons of the survival rates of IPEC-J2 cells and Vero cells, respectively, treated with 100 μg/ml BSN-37 and 100 μg/ml pexiganan at specific four time points. Error bars represent means ± SEM, *n* = 3. *** and **** represents very significance and extremely significance, respectively. (*p* < 0.001 and *p* < 0.0001 by Two-way ANOVA).

In the real-time cell assay, a bidirectional comparison of four groups of two peptides with the same concentration was conducted after the standard cell index (CI) curve was measured. As shown in Figure 7A, all growth curves of IPEC-J2 cells individually treated with five BSN-37 concentrations (50, 100, 200, 400, and 800 μg/ml) reached the confluence compared to untreated cells, which indicates that BSN-37 did not inhibit the growth of IPEC-J2 cells. In contrast, a significant decrease of CI was immediately observed when IPEC-J2 cells were treated with 100 μg/ml pexiganan, and the growth of IPEC-J2 cells was not recovered during the prolonged incubation of 40 h. Conversely, cell survival rates in the group containing 100 μg/ml BSN-37 and pexiganan were 94.75–99.04% and 29.92–38.86%, respectively (shown in Figure 7B). The change tendency of growth curves of Vero cells was similar to that of IPEC-J2 cells when treated by different concentrations of BSN-37 (shown in Figure 7C), which implies that BSN-37 also has no toxicity to Vero cells. The survival rates of Vero cells were 101.1–110.46% in the incubation with 100 μg/ml BSN-37for 40 h (shown in Figure 7D). Pexiganan led to decreased survival rates of Vero cells in the first incubation for about 6 h and then cell growth was recovered step by step with the incubation time. The survival rates of Vero cells were 74.17–105.1% in incubation with pexiganan for 40 h (shown in Figures 7C,D). Collectively, these results indicated that BSN-37 had no toxicity to the tested cells even when the concentration was 800 μg/ml.

### Inhibition Activity of BSN-37 Against Intracellular *S.* Typhimurium

**FIGURE 8:**
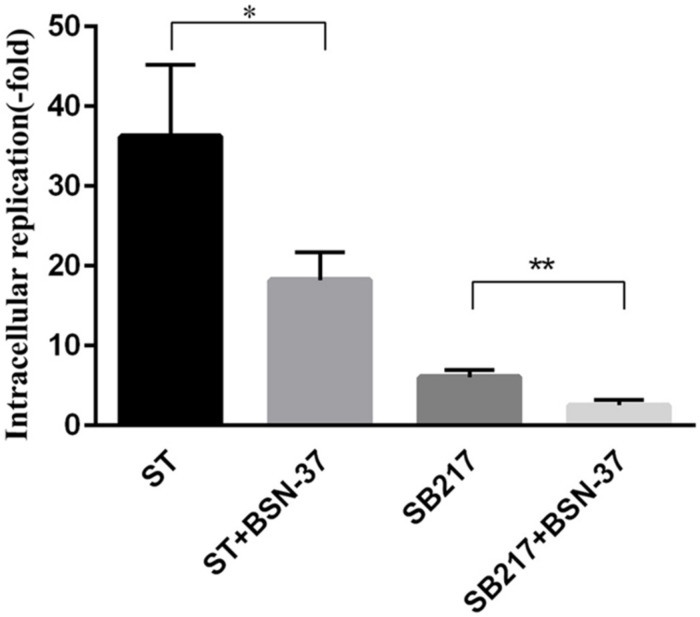
Intracellular replication of ST and SB217 in the presence and absence of BSN-37 in MDCK cells. The concentration of BSN-37 is 400 μg/ml. Error bars represent means ± SEM, *n* = 3. Statistical significance was defined as follows:^∗^*P* < 0.05 and **P < 0.01 (Student’s *t*-test).

In the proliferation inhibition assay, the invasion ability of ST and SB217 against MDCK cells was 0.1 and 0.12%, respectively. After the co-culture for 14 h, the increased fold of intracellular replication of ST and SB217 in MDCK cells is 36.23 ± 5.2 and 6.08 ± 0.5, respectively, (shown in Figure 8). After the MDCK cells harboring ST and SB217 were treated with BSN-37 at the concentration of 400 μg/ml for 14 h, the intracellular replication capacities of these two strains both exhibited significantly decrease compared to that of their corresponding untreated group, which demonstrates that BSN-37 can effectively inhibit the proliferation of intracellular *S.* Typhimurium.

## Discussion

proline-rich peptides contain arginine-rich cationic N-terminal regions followed by more hydrophobic proline-rich repetitions. In aqueous solutions or in lipid vesicles, proline-rich sequences can promote the formation of a left-handed type II polyproline helix that relates to the microbicidal effects of the PRPs (Antonyraj et al., 1998; Otvos, 2002). Alternatively, research has demonstrated that the arginine-rich cationic N-terminal regions also play an important role in the biological activity of PRPs. The structure-activity relationship of Bac5 and Bac7, another PRP from bovine neutrophils, showed that the truncated fragments Bac5(4-23) and Bac7(5-23) lost antibacterial activity when the first three and four amino acid residues were completely removed from their corresponding active fragments, Bac5(1-23) and Bac7(1-23), respectively. The absence of the first arginine residue in the N-terminus of Bac5(1-23) caused a 2-to 4-fold increase in the MICs of Bac(2-23) against *S. aureus* IFO 12 732, *B. subtilis* IFO 3134, and *E. coli* IFO 12734 (Tokunaga et al., 2001; Benincasa et al., 2004). In addition, the N-terminal variant of PR39(1-15) of PR39, a PRP from the porcine small intestine, did not exhibit biological activity when its first three arginine residues were substituted with asparagine (Chan et al., 2001). Furthermore, research showed that the capacity of the two N-terminal residues forming correct H-bonding had a greater effect on the antibacterial activity of Bac7(1-23) than their steric features and local charges (Guida et al., 2015). In this paper, BSN-37 is a fragment of Bac5 from the 2nd residue to the 38th residue, which showed antibacterial activity against the tested strains except for *S. aureus* ATCC25923 (Table 1). The MICs of BSN-37 against *E. coli* ATCC25922 (33.33 μg/ml) exhibited a 2.8-fold increase compared to that of Bac5 (12 μg/ml), which may indicate a lack of the first arginine residue in the N-terminal of BSN-37. However, BSN-37 showed strong antibacterial activity against *S.* Typhimurium (MICs, 8.33–16.67 μg/ml). This activity is similar to that of Bac5 (MICs, 12–25 μg/ml) and the truncated fragment Bac5(1-25) [MICs, 8 μM (about 25 μg/ml)], which has the best antibiotic potency (Gennaro et al., 1989; Mardirossian et al., 2019). This implies that the first arginine residue in Bac5 has no significant effect on its antibacterial activity against *S.* Typhimurium. The antibacterial activity of Bac5 and its truncated fragments exhibit bacterial-specificity, and *S.* Typhimurium seems to be a good target attacked by these peptides.

The lipopolysaccharide (LPS) monolayer on the outer membrane of Gram-negative bacteria prevents antibacterial agents from entering bacteria. Some ions such as Ca^2+^ and Mg^2+^, can stabilize LPS molecules by binding to the negatively charged groups and affecting the antibacterial activity of some drugs (Loh et al., 1984). Most AMPs carry a net positive charge and can interact with LPS carrying a net negative charge (Ding et al., 2003). Therefore, the ions in serum are important factors affecting the antibacterial activity of AMPs *in vivo*. The research demonstrated that the presence of Ca^2+^ or Mg^2+^ with a concentration of 0.5 mM caused a significant reduction in the bactericidal activity of Bac5 and Bac7 against *E. coli* ATCC25922 (Gennaro et al., 1989). QaBac5 and ChBac5 had no activity against *C. albicans* 820 with a high concentration of Na^+^ (100 mM). QaBac5 and CHBac5 also showed similar antibacterial activity against *E. coli* ML-35p and *Listeria monocytogenes* EGD at low and high concentrations of Na^+^. Moreover, ChBac5 showed reduced activity against *S.* Typhimurium14028S at a high concentration of Na^+^ (Shamova et al., 1999). In this study, the physiological concentrations of NaCl, MgCl_2_, and CaCl_2_ significantly decreased the antibacterial activity of BSN-37 against *E. coli* ATCC25922 (increased MICs > 6-fold, Table 2). However, the MICs of BSN-37 against ST did not show remarkable changes in the presence and absence of six salts (increased MICs <1.5-fold) except for CaCl_2_ (increased MICs ≈ 4-fold). These results demonstrate that the effect of some ions on the antibacterial activity of PRPs is concentration- dependent and bacteria-dependent. BSN-37 may keep relatively stable antibacterial activity against *S.* Typhimurium *in vivo*.

Most AMPs are cationic and amphipathic molecules that kill bacteria by disrupting the cell membrane through the formation of ion channels or transmembrane pores. PRPs have been shown to kill susceptible organisms by inhibiting the activity of bacterial cytoplasmic targets without causing significant membrane perturbation (Mishra et al., 2018). QaBac5mini, a truncated fragment of QaBac5, and QaBac7.5mini can facilitate the uptake of NPN when interacting with *E. coli* at the MICs (0.4–0.5 μg/ml). However, QaBac5mini and QaBac7.5mini did not impair the inner membrane integrity above these values (Anderson et al., 2004). Likewise, similar results were observed in the PRPs of ChBac3.4, ChBac5, ChBac7Nα, and ChBac7Nβ (Shamova et al., 2009; Shamova et al., 2016). These results indicate that the PRPs can reach the bacterial inner membrane by increasing the permeability of the outer membrane in gram-negative bacteria, and PRPs likely enter the cytoplasm with the help of membrane proteins. Research demonstrated that Bac7(1-35), Bac5(1-25), and Bac5(1-31) entered *E. coli* by the inner membrane transport proteins, SbmA (Mardirossian et al., 2018; Mattiuzzo et al., 2007). In this study, 50 μg/ml (8-fold MICs) pexiganan caused significant damage in the outer and inner membrane of ST, which agrees with the action mechanism of forming toroidal pores in the bacterial membrane (Gottler and Ramamoorthy, 2009). In contrast, 200 μg/ml (about 12-fold MICs) BSN-37 increased the outer membrane permeability of ST but did not impair the inner membrane integrity. Simultaneously, no broken or ruptured cells were found in the SEM and TEM figures. This strongly supports that BSN-37 conducts antibacterial activity in the cytoplasm rather than disrupting the bacterial inner membrane. However, during the detection of inner membrane integrity a slight increase (about 50 AU) of the fluorescence intensity of DiS-C_3__–_(5) was observed after it was incubated with the treated ST for about 2 min, which may be a consequence of an inner membrane disturbance caused by the BSN-37-mediated inhibition of intracellular targets. A similar phenomenon was also reported in *E. coli* CGSC 4908 treated with an AMP P-Der at 10 × its MIC, which is an amidated hybrid of flounder pleurocidin and frog dermaseptin (Patrzykat et al., 2002). Bac5 can induce respiratory inhibition and drop the ATP content in *S.* Typhimurium (Skerlavaj et al., 1990). Furthermore, Bac5(1-25), Bac5(1-31), and Bac7(1-35) conducts antibacterial activity by binding to the peptide exit tunnel in the bacterial 70S ribosomes (Mardirossian et al., 2018; Seefeldt et al., 2016). Except for the inhibition of protein synthesis, Bac7 could still prevent chaperon-assisted protein folding by binding to the chaperon protein, DanK, and inhibiting the growth of *E. coli* in extremely acidic environments by targeting arginine decarboxylase (Ho et al., 2016). In addition, PR-39 can inhibit protein and DNA synthesis. At the same time, PR-39 also participates in a variety of processes, including the promotion of wound repair, induction of angiogenesis, neutrophils, and chemotaxis, and inhibition of phagocyte NADPH oxidase activity by binding to intracellular SH3-containing proteins (Holani et al., 2016). It is with multiple targets in the cytoplasm that bacteria hardly produce resistance to the PRPs.

Some AMPs not only kill the pathogen but also induce cytotoxicity which restricts their future use in clinical practice. In our study, pexiganan exhibited strong antibacterial activity to the tested strains. Additionally, pexiganan produced strong toxicity to IPEC-J2 cells and Vero cells, which align with our previous results (Zhang et al., 2015). However, BSN-37 did not induce toxicity in the tested cells when its concentration was 800 μg/ml (about 178 μM). Interestingly, the survival rate of all IPEC-J2 cells treated with BSN-37 at the concentration ≥100 μg/ml were over 100%. Research demonstrated that Bac5(1-31) displayed concentration-dependent toxicity against NIH 3T3 cells, which was significant at 32 μM (Mardirossian et al., 2018). Bac5(1-25) caused a 30% decrease of the viability of MEC-1 cells when its concentration was 64 μM (Mardirossian et al., 2019). Moreover, ChBac3.4 at its IC_50_ exhibited toxicity to K-562 cells and U-937 cells but no toxicity to A-549 cells, MRC-5cells, and normal human skin fibroblasts (Shamova et al., 2009). Therefore, the effect of BSN-37 on other eukaryotic cells still need further studied.

BSN-37 exhibits strong activity against *S.* Typhimurium *in vitro* and were non-toxic to the tested eukaryotic cells; therefore, this study investigated the effect of BSN-37 on the inhibition of the proliferation of intracellular *S.* Typhimurium. The results showed that BSN-37 significantly reduced the amount of intracellular *S.* Typhimurium, which suggests that BSN-37 can enter MDCK cells and kill intracellular *S.* Typhimurium. It has been reported that Bac7(1-35) could be rapidly taken into 3T3 and U937 cells through a non-toxic energy- and temperature-dependent process (Tomasinsig et al., 2006). Furthermore, Ba7(1-35) was used to treat the mice infected by *S.* Typhimurium. The peptide significantly increased the number of survivors and the mean survival times of treated mice. Likewise, Ba7(1-35) also remarkably reduced the bacterial load in the organs of treated mice (Benincasa et al., 2010). Unfortunately, no member of Bac5 and its truncated fragments were used to perform animal experiments until now. Although BSN-37 exhibits good characteristics *in vitro* against *S.* Typhimurium, many experiments need to be performed in the future to select a potential candidate for the development of anti-*Salmonella* agents.

## Conclusion

We investigated the characteristics of a novel AMP BSN-37, which showed good antibacterial activity and relative ionic stability against *S.* Typhimurium. BSN-37 at the concentration of 100 μg/ml could kill *S.* Typhimurium after co-incubation for 6 h. The bactericidal mechanism of BSN-37 is a non-lysis action model. Likewise, BSN-37 did not exhibit cell toxicology even at 800 μg/ml and significantly inhibited the proliferation of intracellular *S.* Typhimurium.

## Data Availability Statement

The datasets generated for this study are available on request to the corresponding author.

## Author Contributions

YS and JH conceived and designed the experiments. LY, KZ, BHu, and YC performed the experiments. YX, BHa, and LW contributed reagents, materials, and analysis tools. YS and XX wrote the manuscript.

## Conflict of Interest

The authors declare that the research was conducted in the absence of any commercial or financial relationships that could be construed as a potential conflict of interest.

## Footnotes

1 http://aps.unmc.edu/AP/main.php
